# Characteristics of sudden cardiac arrest during endurance racing: a decade of the Paris registry

**DOI:** 10.1093/europace/euaf313

**Published:** 2026-02-09

**Authors:** Richard Chocron, Thomas Laurenceau, Pierre Cezard, Marion Chabrol, Soline Mignot, Ugo Meli, Camille Langlois, Peter J Schwartz, Stefan Kääb, Bernard I Levy, Frankie Beganton, Wulfran Bougouin, Alain Cariou, Frédéric Adnet, Florence Dumas, Thomas Loeb, Anne-Laure Feral-Piersens, Matthieu Heidet, Daniel Jost, Jean-Philippe Empana, Xavier Jouven

**Affiliations:** Paris Cardiovascular Research Center (PARCC), INSERM, Paris Cité University, 20 rue Leblanc, Paris F-75015, France; Emergency Department, AP-HP, Georges Pompidou European Hospital, Paris F-75015, France; Paris Cardiovascular Research Center (PARCC), INSERM, Paris Cité University, 20 rue Leblanc, Paris F-75015, France; Emergency Department, AP-HP, Georges Pompidou European Hospital, Paris F-75015, France; Paris Cardiovascular Research Center (PARCC), INSERM, Paris Cité University, 20 rue Leblanc, Paris F-75015, France; Emergency Department, AP-HP, Georges Pompidou European Hospital, Paris F-75015, France; Paris Cardiovascular Research Center (PARCC), INSERM, Paris Cité University, 20 rue Leblanc, Paris F-75015, France; Paris Cardiovascular Research Center (PARCC), INSERM, Paris Cité University, 20 rue Leblanc, Paris F-75015, France; Paris Cardiovascular Research Center (PARCC), INSERM, Paris Cité University, 20 rue Leblanc, Paris F-75015, France; Paris Cardiovascular Research Center (PARCC), INSERM, Paris Cité University, 20 rue Leblanc, Paris F-75015, France; Paris Cardiovascular Research Center (PARCC), INSERM, Paris Cité University, 20 rue Leblanc, Paris F-75015, France; Center for Cardiac Arrhythmias of Genetic Origin, Istituto Auxologico Italiano IRCCS, Milan, Italy; Department of Medicine I, University Hospital, LMU Munich, , Munich, Germany; DZHK (German Center for Cardiovascular Research), Partner Site Munich, Munich Heart Alliance (MHA), Munich, Germany; Paris Cardiovascular Research Center (PARCC), INSERM, Paris Cité University, 20 rue Leblanc, Paris F-75015, France; Paris Cardiovascular Research Center (PARCC), INSERM, Paris Cité University, 20 rue Leblanc, Paris F-75015, France; Paris Cardiovascular Research Center (PARCC), INSERM, Paris Cité University, 20 rue Leblanc, Paris F-75015, France; Paris Cardiovascular Research Center (PARCC), INSERM, Paris Cité University, 20 rue Leblanc, Paris F-75015, France; Samu de Paris, Université Paris Cité, hôpital Necker-Enfants malades, Paris, France; Paris Cardiovascular Research Center (PARCC), Université Paris Cité, INSERM U970, European Georges Pompidou Hospital, Paris, France; Paris Cardiovascular Research Center (PARCC), INSERM, Paris Cité University, 20 rue Leblanc, Paris F-75015, France; Samu des Hauts-de-Seine, Assistance Publique-Hôpitaux de Paris, Hôpital Raymond Poincaré, Garches, France; SAMU 93—SMUR—Emergency Department, Avicenne Hospital, Public Assistance Hospitals Paris, Bobigny, France; Sorbonne Paris Nord University, LEPS, UR 3412, Villetaneuse, France; Université Paris-Est Créteil (UPEC), LISSI, EA-3956 (TincNET), Créteil, France; SAMU 94 and Emergency Department, AP-HP, Henri Mondor University Hospital, Créteil, France; Paris Cardiovascular Research Center (PARCC), INSERM, Paris Cité University, 20 rue Leblanc, Paris F-75015, France; Emergency Department, Paris Fire Brigade, Paris, France; Paris Cardiovascular Research Center (PARCC), INSERM, Paris Cité University, 20 rue Leblanc, Paris F-75015, France; Paris Cardiovascular Research Center (PARCC), INSERM, Paris Cité University, 20 rue Leblanc, Paris F-75015, France; Cardiology Department, AP-HP, Georges Pompidou European Hospital, Paris, France

**Keywords:** Cardiac arrest, Endurance races, Sport, Sex differences

## Abstract

**Aims:**

The rising popularity of endurance races underscores the need to explore the risks of sports-related sudden cardiac arrest (Sr-SCA). Although rare, Sr-SCA is significantly more prevalent in men than in women. The mechanisms underlying these sex differences remain unclear.

We aimed to investigate the incidence rates, clinical characteristics, aetiologies, sex differences, and exercise performances among SCA cases during major endurance races in Paris over a 10-year period.

**Methods and results:**

We Analysed the Paris Sudden Death Expertise Centre Registry Data (Covering 2011–2024, excluding 2020). This included SCA cases from the half marathon, full marathon and 20 km Parisian race events. We calculated the incidence rates for men and women, with performance analyses focusing on acceleration patterns and the relative risk of SCA in the final kilometre. Among the 1.2 million participants, 17 SCA cases (88% male) were identified, yielding crude incidences of 16.9 and 5.7 per million for men and women, respectively. Sr-SCA was overrepresented in the final kilometres of short races. Men exhibited twice the acceleration rate that women did. Despite extensive medical investigations, no cause was identified in 47.1% of the cases, underscoring the idiopathic nature of Sr-SCA. After hospitalization, 88% (15/17) of the cases survived, all with excellent neurological outcomes [cerebral performance category (CPC) 1], except for one CPC 2.

**Conclusion:**

SCA incidences during endurance races are low, with male predominance, high survival rates, and a high proportion of unexplained cases. The male-specific acceleration in the final kilometre may suggest that physiological and behavioural factors influence SCA risk.

## Graphical Abstract

**Graphical Abstract:**
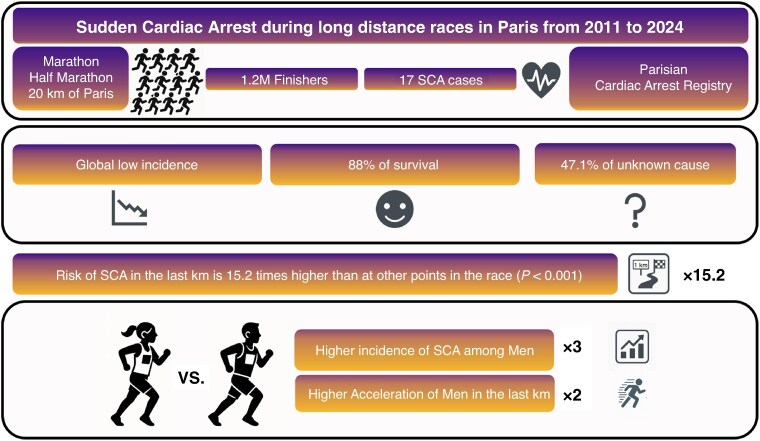

What’s new?

- This is the first large-scale, prospective registry describing sudden cardiac arrest (SCA) during endurance racing in Paris over a 10-year period.
- SCA during races is a rare event, with an incidence ranging from 0.9 to 2.2 per 100,000 finishers
- We identified a clear overrepresentation of SCA in the final kilometre of races, especially in shorter races.
- Male runners displayed a distinct acceleration pattern in the last kilometre, nearly twice as frequent as in women.
- The implementation of an adapted emergency medical system, including strategic prepositioning of medical teams, was associated with excellent survival outcomes (88%).
- These findings provide new insights into sex-specific risk, temporal clustering of events, and practical strategies for optimizing emergency preparedness during endurance races.

## Introduction

The global popularity of long-distance running events has been driven by increasing public awareness of the health benefits of regular exercise. However, cases of sudden cardiac arrest (SCA) occurring during endurance races, often highlighted by extensive media coverage, have raised concerns about the potential health risks associated with this sport. In recent years, the number of women participating in endurance races has increased, with a significant difference in the incidence of SCA between men and women during these events.^1,2^ Two recent studies from the USA and Europe indicated that the incidence of sports-related SCA (Sr-SCA) in women was about 10 times lower than in men across years, even after adjusting for comorbidities and the circumstances of arrest.^1,3^ These disparities remain poorly understood, as there are no clear explanations for this difference^4–6^

Sr-SCA is a specific type of SCA with specific features.^7,8^ Sr-SCA can occur even in young, seemingly healthy individuals, including athletes.^9^ Cardiomyopathies have been consistently implicated as the leading cause of cardiac arrest in young competitive athletes, with hypertrophic cardiomyopathy and arrhythmogenic right ventricular cardiomyopathy accounting for more than one-third of fatal cases.^10^ These findings may not be fully applicable to long-distance runners, who tend to be older and present with different cardiovascular risk factors and pre-existing conditions leading to ischaemic diseases.^11,12^

Several hypotheses, including those theorizing the importance of pathophysiological, metabolic, and morphological differences, have been proposed to explain this sex difference in incidence, but none have been conclusive.^13^ Innate biological mechanisms are increasingly recognized as influential^14^ but remain hypothetical explanations. Despite these findings, no single physiological, biological, or genetic explanation accounts for the low Sr-SCA risk in women.^15^

We hypothesize that analysing the clinical characteristics and exercise performances during endurance races would help to objectively assess sex differences in Sr-SCA incidence and prognosis and to elaborate on sex differences in metabolic and psychological factors during strenuous physical activity. We test this hypothesis using extensive data from major endurance races in Paris over the last 10 years.

## Methods

### Study design and data source

We used two independent data sources, one for SCA cases and one for race participations.

### SCA cases

Every case of unexpected out-of-hospital cardiac arrest in persons older than 18 that occurred between 9 October 2011 and 7 April 2024 in Paris (France) and its inner suburbs (Hauts-de-Seine, Seine-Saint-Denis, and Val-de-Marne) was collected through the Paris Sudden Death Expertise Centre (SDEC). The SDEC registry is a multicentre population-based system covering 6.7 million inhabitants (10% of the French population).^16^ This system records information prospectively and continuously regarding the occurrence (Utstein criteria), management (pre- and in-hospital), and patient outcomes (survival and neurological outcomes) of all SCA cases.^16^ The SDEC registry encompasses the area where the analysed endurance race takes place, enabling the comprehensive collection of all Sr-SCA cases. The study’s inclusion criterion was SCA that occurred during one of the three most prominent races in Paris: The half-marathon (HM), the full marathon (FM), and the 20 km. The exclusion criteria were obvious non-medical causes of SCA following the ILCOR definition.^17^ Each case was thoroughly reviewed to ascertain the exact location of the sudden death within the race and to verify that the individual had indeed participated as a runner rather than as a spectator. SCA cases were considered race-related if they occurred on the same day as the event and within the race time window (up to 6 h after the start for the 20 km and HM, and up to 12 h for the FM), corresponding to the maximum completion times plus a 2-h margin after the last finishers.

### Race participation and performance data

Race participation data on events involving the HM, FM, and 20 km for the 2011–2024 period were collected in open source from the official website (https://www.hokasemideparis.fr/fr/course/resultats). No race was held in 2020 because of the pandemic. We collected the runners’ times over the HM from 2015 to 2019 and from 2021 to 2024. We collected the data year by year before aggregating them. With timing control points placed every 5 km and at the finish line, the HM course (with timing controls at kilometre 5, 10, 15, 20, and 21.097) allowed us to study the acceleration in the last kilometre, unlike the 20 km race (with timing controls at kilometres 5, 10, 15, and 20) and the FM (with timing controls at kilometres 5, 10, 15, 20, 25, 30, 35, 40, and 42.195). We removed data where timing points were missing at kilometres 5, 10, 15, 20, or 21.097 (*n* = 10.921, 3.3% of the total). These missing data might be due to a timing chip malfunction or a participant abandoning the race, but the exact reason cannot be determined. The available data included information on the race finishers of each event, which we exclusively analysed for this study.

### Assessment of medical investigations

Comprehensive investigations included cardiac MRI and pharmacological tests alongside traditional tests, such as echocardiography (ECG) and coronary angiography. Data on other tests, such as electrophysiological study, Holter-ECG, cardiac biopsy, signal-averaged ECG, and genetic tests, were also collected because of their potential to identify specific phenotypes in unexplained SCA cases. The definition of the underlying cause of SCA was based on the results of these standardized investigations, ensuring that case classification relied on consistent diagnostic criteria. These medical investigations for survivors have previously been described by Waldmann et al.^18^

### Statistical analysis

Continuous data are presented as mean ± standard deviation (SD), and categorical data are presented as *n* (%). We compared the data according to sex using a Chi-squared test for categorical variables and a Student’s test for continuous variables.

To calculate the incidence of Sr-SCA, we collected data on all finishing participants across three races and recorded the number of SCA cases. The incidence rate was calculated by dividing the number of Sr-SCA cases by the total number of finishing participants. This was done separately for men and women to assess possible sex differences in incidence rates. The acceleration was calculated by comparing the speed over the last kilometre with the average speed over the previous 5 km, with each person being their own control. The acceleration in the final kilometre could only be calculated for the HM as a result of the last two-timing controls at kilometres 20 and 21.097.

We determined the proportion of SCA cases that occurred in the last kilometre by dividing the number of SCA cases at this point by the total number of cases. Then, we quantified the excess risk of an SCA occurring in the last km. We compared the observed probability of an SCA occurring in the last kilometre (*p*_obs_) with the expected probability (*p*_exp_), assuming a uniform distribution of SCA throughout the race. This comparison allowed us to calculate the relative risk of SCA in the final kilometre of the race and by sex.

To estimate the increased risk of sudden death in the last kilometre of a race, we calculated 95% confidence intervals (95% CIs) using the *z*-score. To estimate the 95% CI for the ratio of men/women in a given acceleration category, we used the log ratio method. *P*-values less than 0.05 were considered statistically significant. All statistical analyses were performed using the open-source Python 3.12.0 software package.

### Ethical considerations

The Paris SDEC registry used for the purpose of this study was approved by the *Commission Nationale de l’Informatique et des Libertés* (Authorization number DR-2012-445). In accordance with the regulations in force, informed patient consent was not required because of the retrospective and observational nature of the study.

## Results

**Figure 1:**
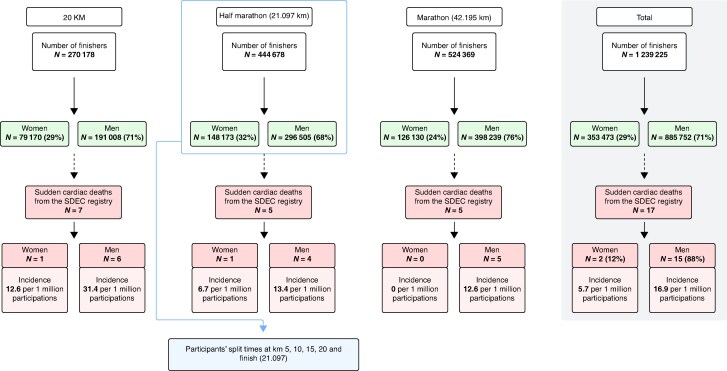
Flow diagram of study participants. Total number of participations in the three races broken down by gender, with the incidence of SD shown for each sub-group. For the half-marathon, performance data was collected at intermediate points at 5, 10, 15, 20 km, and at the finish (21.097 km). The data used for cases of SD and for the census of all participants cover the period from 2011 to 2024, with the exception of 2020. Half-marathon performance data were collected for the years 2015–2024 (with the exception of 2020), the years for which data were publicly available. SNDS, French National Health Insurance; SDEC, sudden death expertise Center; HF, half-marathon; FM, full-marathon.

For the HM and FM events from 2011 to 2024, a total of 969 047 finishers were recorded, representing 599 541 unique participants with a mean age of 37.1 ± 10.8 years at first participation, 61% of whom were men. The Paris 20 km event accounted for an additional 270 178 finishers. The 17 cases of Sr-SCA occurring during the three main races were part of the 405 cases of Sr-SCA registered in the SDEC registry, with no obvious extra-cardiac cause. These 405 cases comprised 21 (5%) women aged 49 ± 20 years and 384 (95%) men aged 51 ± 15 years. These 405 Sr-SCA cases represented 0.9% of the 43 334 SCA cases collected by the SDEC registry over the same period (*Figure 1*).

### Clinical characteristics of SCA

Over the last 10 years and among the 1.2 million participations (mean age: 38 ± 11 years; 71.6% men), there were 17 SCA cases (7, 5, and 5 in the 20 km, HM, and FM, respectively). In these three races, SCA incidence in men was 16.9 cases per million participations (95% CI: 9.31–25.61), and the incidence in women was 5.7 cases per million participations (95% CI: 0.0–14.67).

Of the 17 SCA cases, 88% (15/17) were men, with a mean age of 42 ± 13 years (see Supplementary material online, Table  *S1*). Following the medical evaluation described in *Table 1*, a cause was identified in nine patients: ischaemic cardiomyopathy in five patients (29.4%), one patient with Brugada syndrome, one patient with myocarditis, and one patient with an anatomical abnormality of the right coronary artery. A genetic mutation that may be associated with the cause was identified in one case (a heterozygote mutation on the SCN5A and VCL genes). The cause remained unknown in eight cases (47.1%; *Table 1*).

**Table 1:** Characteristics and clinical assessment of SCA cases during endurance races (20 km of Paris, half-marathon and marathon)

	20 km of Paris							Half Marathon					Marathon				
Patient	1	2	3	4	5	6	7	8	9	10	11	12	13	14	15	16	17
**Gender**	Woman	Man	Man	Man	Man	Man	Man	Woman	Man	Man	Man	Man	Man	Man	Man	Man	Man
**Age at occurrence**	27	53	61	36	34	30	43	32	35	32	60	33	42	48	63	25	36
**Weight**	—	86	—	70	74	—	—	55.3	79	71.5	—	—	75	86	—	80	78.5
**Height**	—	184	—	178	178	—	—	185	176	170	—	—	182	187	—	183	177
**Circumstances of cardiac arrest**																	
**Warning sign**	No	No	No	No	No	—	—	No	No	Yes	Yes	Yes	No	Yes	No	No	No
**Witnessed**	Yes	Yes	Yes	Yes	Yes	Yes	Yes	Yes	Yes	Yes	Yes	Yes	Yes	Yes	Yes	Yes	Yes
**Type of witness**	Bystander with medical skill	Bystander	EMS (ALS or BLS)	Bystander	Bystander with medical skill	Bystander with medical skill	Bystander	Bystander	EMS (ALS or BLS)	Bystander	Bystander	Bystander	EMS (ALS or BLS)	EMS (ALS or BLS)	Bystander	Bystander with medical skill	EMS (ALS or BLS)
**CPR initiated**	Yes	Yes	Yes	Yes	Yes	Yes	Yes	Yes	Yes	Yes	No	Yes	Yes	Yes	Yes	Yes	Yes
**AED applied by bystander**	No	No	Yes	No	No	No	No	No	No	No	No	No	No	No	Yes	Yes	No
**Time response of first EMS team, min**	0:13	0:21	0:10	0:03	—	0:16	—	0:06	—	—	0:12	0:03	0:01	0:30	0:13	0:07	—
**Initial rhythm**	FV	FV	FV	FV	FV	FV	Asystoly	FV	FV	FV	FV	Asystoly	FV	Asystoly	FV	FV	FV
**Initial shock delivered**	Yes	Yes	Yes	Yes	No	Yes	No	Yes	Yes	Yes	Yes	No	Yes	No	Yes	Yes	Yes
**Number of shock**	1	1	1	2	0	2	0	1	2	3	1	0	1	0	1	1	1
**Total dose of epinephrine, mg**	0	0	0	0	0	4	8	0	0	3	0	0	0	0	0	0	0
**Dose of amiodarone, mg**	0	0	0	300	0	0	0	0	0	300	0	0	0	0	0	0	0
**Sustained ROSC**	Yes	Yes	Yes	Yes	Yes	No	No	Yes	Yes	Yes	Yes	Yes	Yes	Yes	Yes	Yes	Yes
**Delay to first CPR, min (noflow)**	0	0	0	0	0	0	0	0	0	2	2	0	0	0	0	0	2
**Duration of CPR, min (Lowflow)**	—	2	5	6	3	52	—	3	4	20	1	10	10	3	7	5	—
**Medical comorbidities**																	
**Any cardiomyopathy**	No	No	No	No	No	No	No	No	No	No	No	No	No	No	No	No	No
**Cancer**	No	No	No	No	No	No	No	No	No	No	Yes	No	No	No	No	No	No
**Thrombo-embolic event**	No	No	No	No	No	No	No	No	No	No	No	No	No	No	No	No	No
**Kidney failure**	No	No	No	No	No	No	No	No	No	No	No	No	No	No	No	No	No
**HIV**	No	No	No	No	No	No	No	No	No	No	No	No	No	No	No	No	No
**Hepatitis virus (B or C)**	No	No	No	No	No	No	No	No	No	No	No	No	No	No	No	No	No
**High blood pressure**	No	Yes	No	No	No	No	No	No	No	No	No	No	No	No	No	No	No
**Diabetes**	No	No	No	No	No	No	No	No	No	No	No	No	No	No	No	No	No
**Dyslipidaemia**	No	No	No	No	No	No	Yes	No	No	No	Yes	No	No	No	No	No	No
**Family history of SCD**	No	No	No	No	Yes	—	—	Yes	Yes	No	No	No	No	No	No	Yes	No
**Smoking**	Never smoking	Previous smoking	Current smoking	Never smoking	Never smoking	Never smoking	Never smoking	Never smoking	Previous smoking	Never smoking	Never smoking	Current smoking	Current smoking	Never smoking	Never smoking	Never smoking	Never smoking
**In hospital interventions**																	
**Interventions**																	
**TTM**	Yes	No	No	Yes	Yes	Yes	No	Yes	Yes	Yes	No	No	Yes	No	No	No	Yes
**ECMO**	No	No	No	No	No	Yes	No	No	No	No	No	No	No	No	No	No	No
**PCI**	No	No	Yes	No	No	—	—	No	No	No	No	Yes	Yes	Yes	Yes	No	No
**Obstructive coronary artery**	—	—	Anterior interventricular artery	—	—	—	—	—	—	—	—	Anterior interventriculae artery	Anterior interventriculae artery	Right coronary artery	Right coronary artery	—	—
**ICD**	Yes	Yes	No	Yes	Yes	No	No	Yes	Yes	Yes	No	No	No	No	No	Yes	Yes
**Diagnostic testing**																	
**CT scan**	Yes	No	Yes	Yes	Yes	No	No	Yes	Yes	Yes	No	No	No	No	Yes	Yes	No
**CT brain scan**	Yes	No	Yes	No	Yes	No	No	Yes	Yes	Yes	No	No	No	No	Yes	No	No
**CT chest scan with contrast**	Yes	No	Yes	No	Yes	No	No	Yes	No	Yes	No	No	No	No	No	No	No
**Coro-rography**	Yes	Yes	Yes	Yes	Yes	No	No	Yes	Yes	Yes	Yes	Yes	Yes	Yes	Yes	Yes	Yes
**Result of coro-rography**	Normal	Normal	Monotroncular	Normal	Normal	—	—	Normal	Normal	Normal	Monotroncular	Monotroncular	Monotroncular	Bitroncular	Bitroncular	Normal	Normal
**Trans thoracic echocardiogram**																	
**Pericardial effusion**	sec	sec	sec	sec	sec	—	—	sec	sec	sec	sec	sec	sec	sec	sec	sec	sec
**Dilated left ventricular**	No	No	No	No	No	—	—	No	No	No	No	No	No	No	No	No	No
**Left ventricular hypertrophy**	No	No	No	No	No	—	—	No	No	No	No	No	No	No	No	No	No
**Right ventricular hypertrophy**	normal	normal	normal	normal	normal	—	—	normal	normal	normal	normal	—	normal	normal	normal	normal	normal
**left ventricular ejection fraction**	>65%	50%	>65%	>65%	>65%	—	—	>65%	>65%	>65%	60%	57%	>65%	>65%	>65%	>65%	60%
**Méthergin test**	Not performed	Negative	Not performed	Not performed	Negative	—	—	Not performed	Not performed	Not performed	Not performed	Not performed	Not performed	Not performed	Not performed	Not performed	Not performed
**Cardiac MRI**	Not performed	normal	Not performed	normal	normal	—	—	normal	myocardite	normal	Not performed	normal	Not performed	Not performed	Not performed	normal	normal
**Ajmaline testing**	Not performed	Not performed	Not performed	Not performed	Not performed	—	—	Not performed	normal	Positive	Not performed	Not performed	Not performed	Not performed	Not performed	normal	normal
**Outcome and diagnosis**																	
**Alive at ICU discharge**	Yes	Yes	Yes	Yes	Yes	No	No	Yes	Yes	Yes	Yes	Yes	Yes	Yes	Yes	Yes	Yes
**Alive at hospital discharge**	Yes	Yes	Yes	Yes	Yes	No	No	Yes	Yes	Yes	Yes	Yes	Yes	Yes	Yes	Yes	Yes
**CPC score at hospital discharge**	1	1	1	1	1	—	—	1	1	1	1	1	1	1	1	1	2
**Fi-l diagnosis**	Unknown	Unknown	Ischemic cardiomyopathy	No ischemic cardiomyopathy	Unknown	Unknown	Unknown	Unknown	No ischemic cardiomyopathy	No ischemic cardiomyopathy	Unknown	Ischemic cardiomyopathy	Ischemic cardiomyopathy	Ischemic cardiomyopathy	Ischemic cardiomyopathy	Unknown	Unknown
**Aetiology of non-ischaemic cardiomyopathy**	—	—	—	A-tomical abnormalities of right coro-ry artery	—	—	—	—	Myocarditis	Brugada syndrome	—	—	—	—	—	—	—

NA, not applicable; EMS, emergency medical services; ALS, advanced life support; BLS, basic life support; ROSC, return of sustained circulation; FV, ventricular fibrillation; CPR, cardiopulmonary resuscitation; TTM, targeted temperature management; SCA, sudden cardiac arrest; CT, computed tomography; PCI, percutaneous coronary intervention: MRI, magnetic resonance imaging; ECMO, extra corporeal membrane oxygenation; ICD, implantable cardioverter defibrillator; CPC, cerebral performance category; ICU, intensive care unit.

The overall survival rate was 88% (15/17), with all women (2/2, 100%) surviving. The two patients who did not survive (Patients 6 and 7) failed to achieve sustained return of spontaneous circulation and remained in refractory cardiac arrest. At hospital discharge, all survivors exhibited excellent neurological outcomes, with all classified as cerebral performance category (CPC) 1, except for one CPC 2 (Patient 17).

### Overrepresentation of sudden deaths in the last kilometre

**Figure 2:**
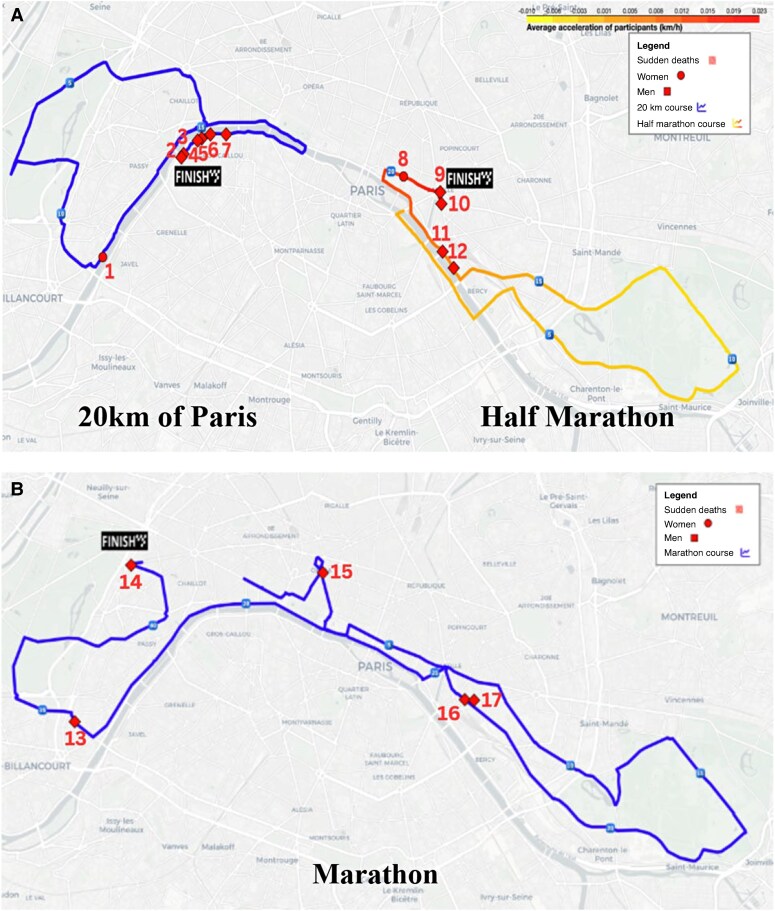
Location of sudden cardiac death on half marathon and 2me0 km (*A*) and on marathon (*B*). Courses of the 20 km, half-marathon (*A*) and marathon (*B*) with location of sudden deaths in red (square for men, circle for women). Acceleration in the last km was available for the half-marathon and is depicted in the map, where yellow denotes no acceleration, while red indicates high acceleration. Continuous speed profiles were obtained by interpolating passage times recorded every 5 km. The acceleration at each point was calculated by measuring the speed differences between consecutive points. For the 20 km and the half-marathon, SCD occurred mainly at the end of the races. In the marathon, no women suffered an SCD. On the marathon route, SCDs are quite evenly distributed along the course (1 at km 3, 2 at km 25, 1 at km 35, and 1 at km 42).

We observed an overrepresentation of SCA in the last kilometre for both the 20 km race and the HM. Specifically, six out of seven SCA cases (only males) occurred in the last kilometre of the 20 km race, and three out of five sudden deaths occurred in the HM. According to our observations in the 20 km and the HM, the risk of experiencing an SCA in the last kilometre is 15.2 (95% CI: 9.2–22.0, *P* < 0.001) times higher than at other points in the race. Two additional SCAs occurred not during but just before the last kilometre in the HM. This pattern was not observed in the FM, where only one out of five SCAs occurred in the last kilometre (*Figure 2*).

### Acceleration patterns in the HM

**Figure 3:**
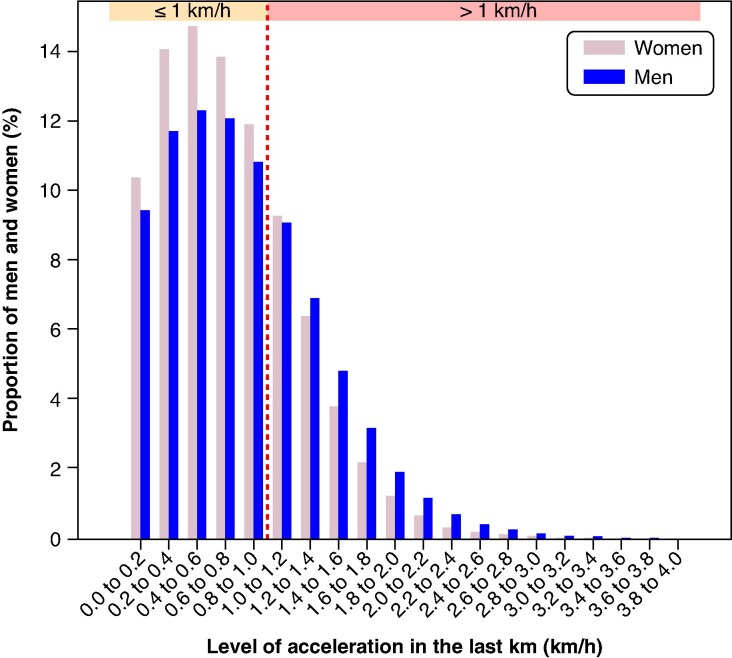
Proportions of men and women by level of acceleration in the last kilometre. Histogram of the proportion of men (blue) and proportion of women (pale pink) by level of acceleration in the last km (in km/h) among the 87% who accelerated. The acceleration was calculated by comparing the speed over the last km with the average speed over the previous 5 km, each person being its own control. The graphic is divided by the red dot line into less than 1 km/h (yellow) and more than 1 km/h (red).

The analysis of performance data focused on 323 028 finishers of the HM, the only race for which acceleration in the final kilometre was available. An acceleration in speed was observed in 87% of the finishers at the end of the race; this acceleration was more pronounced during the last kilometre. The proportion of men who accelerated within 0 and 1 km/h was lower than that of women (0.87 [95% CI: 0.86–0.87], *P* < 0.001). In contrast, the proportion of men who accelerated by more than 2 km/h was nearly twice as high (1.96 [95% CI: 1.86–2.06], *P* < 0.001) as that of women (*Figure 3*).

**Figure 4:**
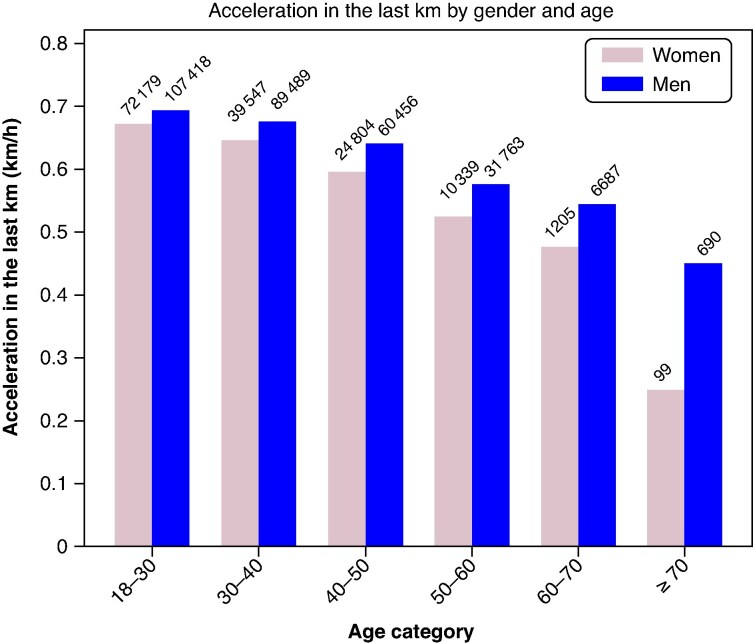
Acceleration in the last km by gender and age. Histogram of the acceleration of men (blue) and women (pale pink) in the last km according to their age category. Acceleration was calculated by comparing the speed over the last km with the average speed over the previous 5 km, each person being their own control.

We assessed acceleration in the last kilometre according to age categories. Across all age groups, on average, men accelerated more than women did (*Figure 4*). Furthermore, the acceleration gap widened with age. The mean acceleration during the last kilometre observed among elites (top 100) was less than 1 km/h: 0.76 ± 0.82 km/h for men and 0.67 ± 0.58 km/h for women (*P* = 0.01).

## Discussion

This study provided new insights into the characteristics, clinical findings, and understanding of sex differences in Sr-SCA during endurance racing. In this large database of more than 1 million participations with extensive SCA information, we confirmed a low incidence of SCA during endurance racing and an excellent prognosis with an overall survival rate of 88%. Despite extensive in-hospital medical assessments, 47.1% of cases remained without a determined aetiology, emphasizing the challenges of identifying the underlying causes of SCA in this context. We observed a significant overrepresentation of male cases. We also noted that Sr-SCA events were predominantly concentrated in the last kilometres, with sex differences. Behavioural responses to acceleration in the last kilometre were twice as high in men as in women. These results may reflect a difference in self-determination in racing strategies between men and women, which may play a key role in the risk of SCA during this critical phase of the race.

The overall survival rate of 88% and 93% CPC 1 at hospital discharge highlights the favourable prognosis associated with immediate medical intervention. This outcome may be attributed to a comprehensive and immediate pre-hospital management strategy, as well as to the healthy, physically active, and relatively young participant population. Notably, the observed survival rate exceeds that reported in other public settings, such as train stations,^19^ airports,^20–22^ and casinos.^23^ While it remains uncertain whether these results can be generalized to other cases of SCA related to sports activities, this study demonstrates that, under these specific conditions, a very high survival rate can be achieved. Similarly, in the USA, the survival from SCA during long-distance races has improved from 29% in 2000–2009 to 66% between 2010 and 2023.^3^ Moreover, this study has observed that SCA occurred in the last quarter of long-distance races.^3^ Our findings provide strong proof of concept supporting the targeted implementation of specialized emergency teams in high-risk zones. Positioning a dedicated medical team in the final kilometres of long-distance races should now be highly recommended.

Beyond the impact of organizational factors on survival, these sex-based findings also suggest that several factors, both physiological and behavioural, may contribute to the increased risk of SCA in male endurance athletes compared to their female counterparts.^24^

Numerous studies have demonstrated that male athletes face a two- to ten-fold higher SCA compared to their female counterparts.^1,10,25–29^ The reasons for this sex-based disparity remain incompletely understood, but several potential protective factors have been proposed for female athletes. These include hormonal influences,^30,31^ sex-specific variations in the expression of cardiovascular disease, differing susceptibilities to arrhythmic triggers,^32^ and distinct patterns of cardiac remodelling in response to athletic training.^29^ Notably, men and women exhibit marked differences in cardiovascular adaptation to endurance training, which may contribute to their respective risks of SCA. Endurance training typically leads to cardiac remodelling characterized by increased heart size and stroke volume, enhancing oxygen delivery during intense physical exertion. In male athletes, these changes manifest as ‘physiological’ cardiac hypertrophy, marked by a 12% increase in left ventricular mass, a 15–20% thickening of the ventricular wall, and a 10% enlargement of the ventricular cavity.^33,34^ While these adaptations support athletic performance, they may also increase the vulnerability of male athletes to SCA, especially under extreme physical stress.^35,36^ By contrast, female athletes tend to develop smaller heart dimensions even with similar endurance training. Although their heart rate during maximal exercise may match that of males, women generally exhibit a lower left ventricular mass and reduced maximal cardiac output.

Recent work further supports the concept that athletic cardiac remodelling may be associated with distinct pro-arrhythmogenic phenotypes, some of which appear to be exercise-induced. In this context, the need for surrogate markers has been highlighted, given that clinical arrhythmic events such as SCA are too rare to be adequately captured in most sports cardiology studies. Moreover, the addition of advanced techniques, such as electroanatomic mapping during electrophysiological studies, has been proposed as a means to identify hidden arrhythmogenic substrates in athletes.^37^ These insights align with our findings of a high proportion of idiopathic cases despite extensive investigations, underlining the persistent challenge of fully characterizing arrhythmic risk in endurance athletes.

In our study, the only case of SCA related to Brugada syndrome occurred immediately after crossing the finish line. This suggests that the event was triggered not by maximal physical effort but rather by the abrupt cessation of exercise, which, incidentally, is the most common pattern with sudden death among athletes: They have a cardiac arrest a few seconds after having stopped running. The underlying mechanism is that cessation of exercise leads to a massive vagal reflex, releasing acetylcholine at a time when circulating catecholamines are still very high. The consequence is an increased dispersion of ventricular refractoriness, and the increased dishomogeneity of repolarization favours re-entrant arrhythmias and ventricular fibrillation.^38,39^ Because exercise training increases the propensity for strong vagal reflexes,^40^ patients with genetic disorders, such as Brugada syndrome and long QT syndrome type 1 (LQT1), whose life-threatening arrhythmias are triggered by high vagal tones and reflexes, particularly when coupled with high sympathetic activity, should avoid or limit exercise training.^41^

Although purely hypothetical, psychological factors might help interpret our findings and warrant further consideration. Research on behaviour in sports highlights both intrinsic factors (e.g. personality) and extrinsic ones (e.g. social or environmental influences) as key motivators during exertion phases.^42,43^ In our study, SCA incidents concentrated in the final kilometre, where male participants were twice as likely as females to accelerate. This difference may reflect distinct pacing strategies influenced by motivation. An alternative explanatory framework is that men may be more prone to risk-taking and overestimating their capacities, while women may adopt more conservative approaches, even when performance levels are similar. This has been observed across domains such as academics, work, and other sports.^44–46^ While no causal link can be established, these psychological patterns offer a thought-provoking lens for future exploration.

### Limitations

Our study has several limitations. First, our work is only descriptive and focuses on rare events. However, we collected all cases within a specific region and time frame, ensuring the exhaustiveness of SCA cases and providing a comprehensive overview of this population. Second, as this is an observational study, it does not establish causality. The small number of SCA cases, reflecting the rarity of the event, limits the robustness of subgroup analyses, particularly sex-specific comparisons. However, the fact that a large proportion clustered in the final kilometre suggests a non-random distribution; nevertheless, these estimates should be interpreted with caution given their limited statistical precision. Although it is often suggested that the intensity of acceleration during races contributes to cardiac arrest,^47,48^ we lack data on the participants’ training routines or the total kilometres completed, as some individuals succumbed before finishing the race. As a sensitivity check, if the number of starters were used instead of finishers, and with a mean percentage of race abandonments around 9.8%, the estimated incidence would be slightly lower. In addition, important covariates such as training load and environmental conditions were not available in our dataset, which may have influenced both performance metrics and clinical outcomes. This limitation may be addressed in the future through the use of connected watches that could potentially provide critical data. We acknowledge that we have been disappointed by the absence of identifiable causes despite performing all currently available medical examinations, highlighting the idiopathic nature of many cases of Sr-SCA, which may be explained by the limitations of current technologies. Not all patients underwent the same extended set of investigations, as certain tests were performed only when clinically indicated. This heterogeneity may have influenced the proportion of cases classified as idiopathic. Moreover, in France, autopsy is not a routine practice and is performed only under specific circumstances, such as upon request from the family or through judicial requisition. In the two fatal cases reported, no autopsy was performed. We acknowledge that autopsy findings could have provided valuable insights into the underlying cause. Although cause determination was not performed by independent blinded reviewers, the risk of misclassification bias is limited since patients were managed in expert centres applying international recommendations for SCA evaluation. These results contribute to the current debate about screening strategies (questionnaires, general practitioner examinations, or ECGs) before sporting exercises and, by extension, the proposed approaches to mitigate the occurrence of SCA. Furthermore, our study was limited to large-scale races, excluding smaller events that could offer a broader perspective.

## Conclusion

This study highlights the low incidence of SCA during endurance racing over a 10-year period in Paris, with male predominance, high survival rates, and a high proportion of unexplained cases. The observed male-specific acceleration in the final kilometres suggests that both physiological and behavioural factors influence Sr-SCA risk.

## Supplementary Material

euaf313_Supplementary_Data

## Data Availability

The data that support the findings of this study are available from the corresponding author [RC] upon reasonable request.
