# Supplementary material for: Characteristics of sudden cardiac arrest during endurance racing: a decade of the Paris registry
Source: Europace. 2026 Feb 9;28(2):euaf313. doi: 10.1093/europace/euaf313 (PMC12884668; doi:10.1093/europace/euaf313)
Supplement: euaf313_Supplementary_Data [file euaf313_supplementary_data.docx]

**Supplemental table 1.** **Utstein characteristics of all SCA during endurance race.**

*Footnote: EMS = Emergency Medical Services: ALS = Advanced Life Support; BLS=Basic Life Support; ROSC=Return Of Sustained Circulation; FV=Ventricular Fibrillation; CPR = Cardiopulmonary resuscitation; TTM =Targeted Temperature Management; SCA = Sudden Cardiac Arrest; CT=Computed Tomography; PCI= Percutaneous coronary intervention: MRI = Magnetic Resonance Imaging; ECMO= Extra Corporeal Membrane Oxygenation; ICD= implantable cardioverter defibrillator; CPC= Cerebral Performance Category; ICU= Intensive Care Unit.*

|  | **Overall** |
| --- | --- |
|  | **n=17** |
| Age, mean(SD) | 41.58(13.4) |
| Woman, n(%) | 2(11.8) |
| Running races in Paris, n(%) |  |
| 20 km | 6(34.3) |
| Full Marathon | 5(29.4) |
| Half marathon | 6(34.3) |
| **Circumstances of cardiac arrest** | |
| Witnessed, n(%) | 17(100.0) |
| Type of witness, n(%) |  |
| Bystander | 8(47.0) |
| Bystander with medical skills | 4(23.5) |
| EMS (BLS or ALS) | 5(29.4) |
| Bystander CPR, n(%) | 15(88.2) |
| AED applied by bystander, n(%) | 3(17.6) |
| Initial schockable rhythm, n(%) | 13(76.5) |
| CA to CPR, minutes (mean (SD)) | 1.25(0.68) |
| Out of hospital outcome |  |
| Refractory cardiac arrest | 2(11.8) |
| ROSC | 15(88.2) |
| **Medical Comorbidites** | |
| Any caridiomyopathy, n(%) | 1(5.9) |
| Cancer, n(%) | 1(5.9) |
| Thrombo-embolic event, n(%) | 1(5.9) |
| Kidney failure (KF), n(%) | 1(5.9) |
| High blood pressure (HBP), n(%) | 1(5.9) |
| Diabetes, n(%) | 1(5.9) |
| Dyslipidemia, n(%) | 2(11.8) |
| Smoking status |  |
| Curent smoking, n(%) | 3(17.6) |
| Never smoking, n(%) | 11(64.7) |
| Previous smoking, n(%) | 2(11.8) |
| **In hospital therapy** | |
| Target Temperature management therapy, n (%) | 8(47.1) |
| CT scan, n(%) | 10(58.8) |
| Extracorporeal life support, n(%) | 1(5.9) |
| Percutaneous Coronary Intervention, n(%) | 15(88.2) |
| Lesion PCI |  |
| Monotroncular lesion | 5(29.4) |
| Bitroncular lesions | 2(11.8) |
| Normal | 8(47.1) |
| Angioplasty, n(%) | 6(35.3) |
| Implantable cardioverter-defibrillator, n(%) | 7(41.2) |
| **Outcomes** | |
| Alive at Hospital discharge, n(%) | 14(82.4) |
| CPC 1-2 at hospital discharge, n(%) | 14(82.4) |
| Alive at 1 year | 14(82.4) |
| Aetiology, n(%) |  |
| Non ischemic cardiopathy | 3(17.6) |
| Unknown | 7(41.2) |
| Ischemic cardiopathy | 7(41.2) |
